# NTAN1 Promotes Glioblastoma Malignant Progression and Is a Novel Prognostic Factor of Poor Prognosis

**DOI:** 10.3390/biomedicines14081870

**Published:** 2026-08-21

**Authors:** Jian Yang, Zihan Lin, Zhihan Wang, Shukai Lin, Minglei Chen, Hao Xu, Qi Lv, Li Gao, Jianwei Ge

**Affiliations:** 1 Department of Neurosurgery, Renji Hospital, Shanghai Jiaotong University School of Medicine, Shanghai 200127, China; 2 State Key Laboratory for Conservation and Utilization of Subtropical Agro-Bioresources, College of Life Science and Technology, Guangxi University, Nanning 530004, China; 3 Department of Neurosurgery, Shanghai Pudong Hospital, Fudan University Pudong Medical Center, 2800 Gong Wei Road, Shanghai 201399, China; 4 Department of Neurosurgery, Sanya Central Hospital (The Third People’s Hospital of Hainan Province), Sanya 572000, China; 5 Department of Neurology, Sanya Central Hospital (The Third People’s Hospital of Hainan Province), Sanya 572000, China; 6 Department of Medical Imaging, Shanghai Xuhui Central Hospital, 366 Longchuan North Road, Xuhui District, Shanghai 200237, China; 7 Department of Neurology, Renji Hospital, Shanghai Jiaotong University School of Medicine, Shanghai 200025, China

**Keywords:** glioblastoma, prognosis, invasive, growth, NTAN1

## Abstract

**Background/Objectives:** Glioblastoma (GBM) is the most aggressive and lethal primary brain tumor, and elucidating the molecular determinants of its malignant progression is essential for improving patient outcomes. NTAN1 encodes N-terminal asparagine amidase 1, a component of the N-degron pathway involved in selective protein turnover; however, its role in GBM remains unclear. This study aimed to investigate the clinical significance and biological function of NTAN1 in GBM. **Methods:** Integrated analyses of The Cancer Genome Atlas (TCGA) transcriptomic and clinical data were performed, together with tissue microarray validation, to assess the association between NTAN1 expression and prognosis in GBM. Immunohistochemical analysis of GBM specimens was conducted to evaluate NTAN1 protein expression. Functional studies, including NTAN1 knockdown and overexpression experiments, were used to examine its effects on GBM cell proliferation, clonogenic growth, migration, and invasion. An orthotopic GBM model was established to assess the effect of NTAN1 inhibition on tumor growth. Transcriptomic analysis was further performed to explore the molecular changes associated with NTAN1 knockdown. **Results:** Elevated NTAN1 expression was associated with poor survival in GBM. Immunohistochemical analysis further demonstrated that high NTAN1 protein expression predicted unfavorable prognosis. Functional studies showed that NTAN1 knockdown inhibited GBM cell proliferation, clonogenic growth, migration, and invasion, whereas NTAN1 overexpression promoted these malignant phenotypes. In an orthotopic GBM model, NTAN1 inhibition significantly reduced tumor volume. Transcriptomic analysis showed that NTAN1 knockdown was associated with reduced expression of invasion-related genes, including *SPINK1*, *MMP1*, and *LIF*, and with enrichment changes in inflammation-associated pathways, suggesting that NTAN1 may promote GBM progression through pro-invasive molecular programs. **Conclusions:** Collectively, these findings identify NTAN1 as a potential promoter of GBM malignant progression and a prognostic biomarker of poor outcome.

## 1. Introduction

Glioblastoma (GBM) is the leading primary malignant tumor of the adult central nervous system, distinguished by its aggressive clinical and biological features [1,2]. Although patients are currently treated with maximal safe resection followed by radiotherapy and temozolomide-based chemotherapy, survival remains limited, with a median overall survival of approximately 15 months [1]. The persistently unfavorable prognosis of GBM is largely attributable to its marked intratumoral heterogeneity, rapid proliferative capacity, diffuse infiltration into surrounding brain tissue, and high recurrence rate [2,3,4,5]. In particular, the infiltrative growth pattern of GBM severely limits complete surgical excision and contributes to inevitable tumor recurrence, representing a major obstacle to durable therapeutic response [6,7]. Therefore, identification of novel molecular determinants involved in GBM progression is of considerable importance for improving prognostic stratification and developing targeted therapeutic strategies.

Advances in large-scale genomic, transcriptomic, and epigenomic profiling have substantially improved our understanding of GBM biology and revealed multiple molecular alterations associated with tumor progression [3,4,5,8,9]. Integrative analyses from The Cancer Genome Atlas (TCGA) and other cohorts have identified key dysregulated pathways involving receptor tyrosine kinase signaling, p53, RB, and metabolic reprogramming, as well as clinically relevant molecular subclasses and epigenetic states [3,4,8,9,10]. More recent single-cell and spatial transcriptomic analyses have revealed extensive cellular plasticity in GBM, as well as dynamic communication between tumor cells and the surrounding microenvironment, including immune cells, stromal compartments, and extracellular matrix remodeling processes, which are implicated in treatment resistance and invasive progression [11,12,13,14]. However, many candidate genes identified through bioinformatics analyses remain insufficiently characterized experimentally, and their clinical significance at the protein level is often unclear. Thus, integrated studies combining survival analysis, functional assays, and tissue-based validation are needed to establish the biological relevance of these molecules in GBM.

NTAN1 encodes N-terminal asparagine amidase 1, a component of the N-degron pathway that contributes to selective protein degradation by catalyzing the deamidation of N-terminal asparagine residues [15,16]. The N-degron pathway, formerly known as the N-end rule pathway, links the in vivo half-life of a protein to the identity of its N-terminal residue and plays a fundamental role in protein quality control, stress responses, and regulation of diverse biological processes [15,16,17,18]. Dysregulation of protein turnover pathways, including ubiquitin-proteasome-dependent proteolysis, has been increasingly implicated in tumor initiation and progression [19,20]. Nevertheless, compared with other components of proteostasis networks, the role of NTAN1 in human malignancies, particularly in GBM, remains poorly understood. To date, the expression pattern, prognostic relevance, and functional significance of NTAN1 in GBM have not been systematically investigated.

In the present study, NTAN1 was selected for further investigation because the preliminary analysis of the TCGA GBM cohort identified it as a candidate gene associated with poor survival, and its role in GBM has not been characterized previously. We hypothesized that elevated NTAN1 expression would contribute to GBM malignant progression and would be associated with unfavorable prognosis. To test this hypothesis, we combined survival analysis, tissue microarray-based immunohistochemical validation, in vitro gain- and loss-of-function assays, in vivo orthotopic modeling, and transcriptomic profiling. Because the downstream pathways regulated by NTAN1 in GBM remain unclear, transcriptomic analysis was further performed to explore molecular programs potentially associated with NTAN1-mediated malignant behavior.

## 2. Material and Methods

### 2.1. Survival Analysis Using the TCGA Dataset

Transcriptomic profiles together with corresponding clinical information for patients with GBM were obtained from The Cancer Genome Atlas database. According to their NTAN1 mRNA abundance, GBM patients were assigned into high- or low-NTAN1 expression groups. The overall survival was assessed using Kaplan–Meier analysis, and differences in survival between the two groups were evaluated with the Mantel–Haenszel test.

### 2.2. Human GBM Tissue Samples

This study received approval from the Ethics Committee of Renji Hospital, School of Medicine, Shanghai Jiao Tong University, under approval number RA-2022-032. A total of sixty-one formalin-fixed paraffin-embedded specimens from patients with primary GBM were retrospectively collected from Renji Hospital, Shanghai Jiao Tong University. These patients were treated between January 2005 and December 2019. Follow-up data corresponding to these cases were collected.

### 2.3. Quantitative Real-Time PCR

Total RNA was extracted from A172, U87, U251, T98G, and U118MG cells using TRIzol reagent (cat. no. 15596026CN; Thermo Fisher Scientific, Waltham, MA, USA), according to the manufacturer’s instructions. cDNA was synthesized from total RNA using HiScript II RT SuperMix (cat. no. R223-01; Vazyme, Nanjing, China). Quantitative real-time PCR (qRT-PCR) was performed on a LightCycler 480 System (Roche, Basel, Switzerland) using SYBR Green Master Mix (cat. no. A25742; Thermo Fisher Scientific, Waltham, MA, USA). The thermal cycling conditions were as follows: initial denaturation at 95 °C for 60 s, followed by 40 cycles of denaturation at 95 °C for 15 s and annealing/extension at 60 °C for 60 s with fluorescence acquisition. Melting curve analysis was subsequently performed from 60 to 95 °C with continuous fluorescence collection. GAPDH was used as the internal reference gene. The Ct values of NTAN1 were normalized to those of GAPDH, and the relative NTAN1 mRNA expression was calculated using the 2^−Ct method (ΔCt = Ct_NTAN1 − Ct_GAPDH). All reactions were performed in triplicate. The primer sequences were as follows: NTAN1 forward, 5′-CATTGTGACGGGAACCGACACCA-3′, and reverse, 5′-TGTCTCGTCACTGAAGCCTCCA-3′; GAPDH forward, 5′-GTCTCCTCTGACTTCAACAGCG-3′, and reverse, 5′-ACCACCCTGTTGCTGTAGCCAA-3′.

### 2.4. Cell Culture

The human GBM cells U87, A172, U251-MG, U118MG, and T98G were obtained from the Shanghai Institute of Cell Biology, Chinese Academy of Sciences. Cells were maintained in modified Eagle’s medium (cat KGL1602, KeyGEN, Nanjing, China), containing 10% fetal bovine serum (cat 10100147, Gibco, Carlsbad, CA, USA), 100 units/mL penicillin, and 100 μg/mL streptomycin. All cells were kept at 37 °C under humidified conditions with 5% CO_2_. These cell lines were authenticated by STR profiling and were verified to be mycoplasma-free.

### 2.5. Lentiviral Infection, Plasmid Construction, and Transfection

Lentiviruses designed for NTAN1 knockdown or overexpression were generated by Hanyin Biotech, Shanghai, China. NTAN1-knockdown lentiviruses were used to infect U87 and A172 cells, whereas NTAN1-overexpression lentiviruses were introduced into T98G and U251-MG cells. The short hairpin RNA sequences directed against NTAN1 were as follows: KD1, 5′-ATTATGTGTGACAGAATTA-3′; KD2, 5′-GACCAATGATTAGCATTA-3′; and KD3, 5′-CACTTCCAGTAATATG-3′.

### 2.6. Cell Proliferation Assay

Cell proliferation was evaluated using the CCK-8 assay. Cells were plated into 96-well plates at a density of 2000 cells per well. On days 1, 2, 3, 4, and 5 after seeding, each well received 10 μL of CCK-8 reagent (Kumamoto Prefecture, cat CK04-01, Kumamoto, Japan). The absorbance was subsequently measured at 450 nm. Each experiment was independently repeated at least three times.

### 2.7. Colony Formation Assay

For colony formation analysis, cells were seeded into 6-well plates at 1000 cells per well. After 9 days of incubation, colonies were fixed and stained using 0.1% crystal violet. Only colonies containing more than 50 cells were included in the counts. All assays were carried out in triplicate.

### 2.8. Transwell/Matrigel–Transwell Assay

The migratory and invasive abilities of GBM cells were assessed using Transwell chambers with 8 μm pore size membranes (LABSELECT, cat. no. 14341, Beijing, China). For the migration assay, 5,000 cells suspended in 200 μL medium containing 2% FBS were seeded into the upper chamber, while 600 μL of complete medium containing 10% FBS and 1% penicillin/streptomycin was added to the lower chamber as a chemoattractant. After incubation for 48 h, cells remaining on the upper surface of the membrane were gently removed with a sterile cotton swab. Cells that had migrated to the lower surface were fixed with 4% tissue cell fixative for 15 min, stained with 0.1% crystal violet for 15 min, and counted under a light microscope at ×200 magnification. Five random fields were evaluated for each well, and the mean number of migrated cells was calculated. All experiments were repeated three times.

For the invasion assay, the same procedure was performed, except that the upper chamber membranes were precoated with 50 μL of Matrigel (BD Biosciences, cat. no. 356234, Shanghai, China) diluted 1:8 before cell seeding. Briefly, 5000 cells in 200 μL of medium containing 2% FBS were added to the upper chamber, and 600 μL of complete medium containing 10% FBS and 1% penicillin/streptomycin was added to the lower chamber. After incubation for 48 h, non-invading cells on the upper surface were removed with a sterile cotton swab. Cells that had invaded through the Matrigel-coated membrane were fixed with 4% tissue cell fixative for 15 min, stained with 0.1% crystal violet for 15 min, and counted under a light microscope at ×200 magnification. Five random fields were evaluated for each well, and the mean number of invading cells was calculated. All experiments were repeated three times.

### 2.9. Xenograft GBM Animal Model

All animal experiments were approved by the Institutional Animal Care and Use Committee of Renji Hospital, Shanghai Jiao Tong University (approval number B-2019-003). Orthotopic GBM xenograft models were established using six-week-old male athymic nu/nu mice purchased from Lingchang Biotech, Shanghai, China. U87 control cells, NTAN1-knockdown cells, or NTAN1-overexpression cells were suspended at 5 × 10^5^ cells in 5 μL for implantation. Using a stereotactic frame, David Kopf Instruments, the prepared cells were stereotactically injected into the corpus striatum of anesthetized nude mice. MRI scanning was performed once body weight loss or neurological symptoms were observed. The tumor size was measured on MRI images, and the tumor volume was determined using Function Analysis software (General Electric, Boston, MA, USA) according to the formula: V = (L × W^2^)/2. Animals were sacrificed upon the appearance of severe emaciation or depressive behavior. Animal housing and all surgical procedures were conducted in accordance with the Guide for the Care and Use of Laboratory Animals.

### 2.10. Transcriptome Sequencing

Transcriptome sequencing was performed by GENEWIZ Biotech, Suzhou, China. Total RNA was extracted from samples using TRIzol Reagent following the manufacturer’s protocol. The remaining genomic DNA was removed by treatment with DNase I (Takara, Beijing, China). RNA quality and integrity were evaluated using an Agilent 2100 Bioanalyzer, while the RNA concentration was measured using the ND-2000 spectrophotometer (NanoDrop Technologies, Wilmington, DE, USA). RNA-seq libraries were generated from 1 μg of total RNA with the VAHTS^®^ Universal V8 RNA-seq Library Prep Kit for Illumina, San Diego, CA, USA. After quantification with TBS380, paired-end sequencing was carried out on the Illumina NovaSeq 6000 platform using a read length of 2 × 150 bp.

### 2.11. Immunohistochemistry

Immunohistochemical staining was performed on 5 μm-thick paraffin-embedded human tumor tissue sections. After deparaffinization and rehydration, endogenous peroxidase activity was blocked with 3% hydrogen peroxide. Antigen retrieval was carried out in sodium citrate buffer (pH 6.0) at 100 °C for 20 min. The sections were then blocked with 5% bovine serum albumin at 37 °C for 1 h and incubated with anti-NTAN1 primary antibody (PNAA Rabbit pAb; Bioss, cat. no. bs-19495R; 1:400, Beijing, China) at 4 °C overnight. After washing three times with PBST for 5 min each, the sections were incubated with S-vision polymer secondary antibody (Servicebio, cat. no. G1313, Wuhan, China) at room temperature for 30 min. Immunostaining was visualized using the S-vision immunohistochemical kit (Servicebio, cat. no. G1313, Wuhan, China) with DAB as the chromogen, followed by hematoxylin counterstaining. IgG was used as the negative control. Images were acquired using an Olympus CKX-41 microscope at ×200 magnification.

### 2.12. Statistical Analysis

Statistical analyses were performed using SPSS version 17.0 (SPSS Inc., Chicago, IL, USA) and GraphPad Prism 9.5.1 (GraphPad Software, San Diego, CA, USA), where appropriate. Kaplan–Meier survival curves were compared using the log-rank test. Cox proportional hazards regression analysis was performed to assess the association between NTAN1 expression and survival outcomes. Comparisons between two groups were performed using a two-tailed Student’s *t*-test. For experiments involving more than two groups or multiple time points, one-way or two-way ANOVA followed by appropriate post hoc tests was used, as applicable. Data are presented as the mean ± SD from at least three independent experiments. A *p*-value < 0.05 was considered statistically significant.

## 3. Results

### 3.1. Elevated NTAN1 Expression Is Associated with Poor Prognosis in GBM

**Figure 1:**
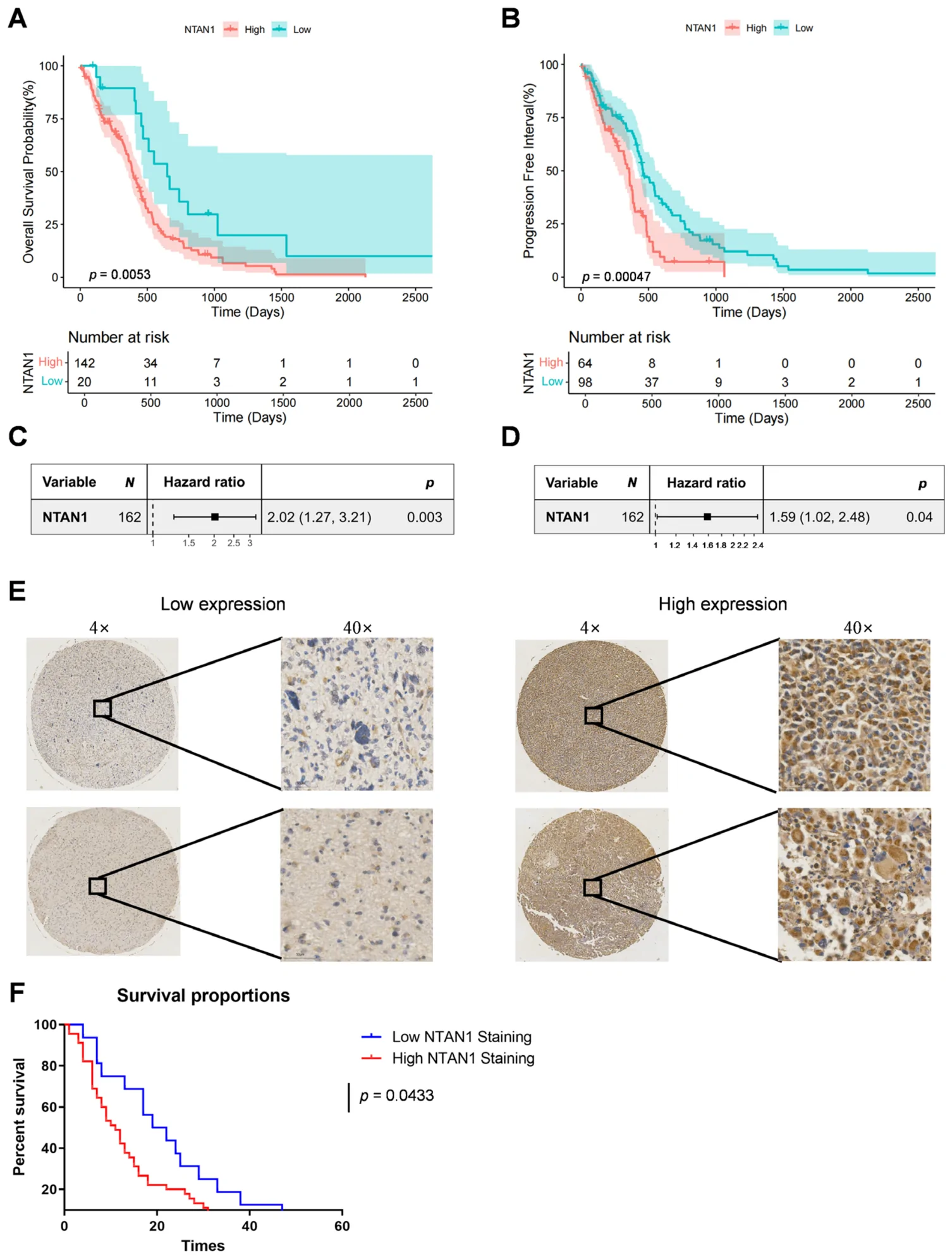
High NTAN1 expression is associated with unfavorable survival outcomes in GBM. (**A**) Kaplan–Meier overall survival (OS) analysis of patients with GBM from the TCGA database stratified by NTAN1 expression. (**B**) Kaplan–Meier progression-free interval (PFI) analysis of patients with GBM from the TCGA database stratified by NTAN1 expression. (**C**) Cox proportional hazards analysis of NTAN1 expression as a continuous variable for OS. (**D**) Cox proportional hazards analysis of NTAN1 expression as a continuous variable for PFI. (**E**) Representative immunohistochemical staining images of NTAN1 expression in GBM tissue microarray samples, showing low and high NTAN1 expression. NTAN1 staining was predominantly localized in the cytoplasm of tumor cells. (**F**) Kaplan–Meier overall survival analysis of GBM patients in the tissue microarray cohort according to NTAN1 protein expression levels.

The prognostic relevance of NTAN1 expression in GBM was first analyzed using data from TCGA. The Kaplan–Meier survival analysis showed that patients with high NTAN1 expression had a significantly reduced overall survival (OS) compared with those with low NTAN1 expression (log-rank *p* = 0.0053) (Figure 1A). Similarly, patients in the high-NTAN1 expression group exhibited a significantly shorter progression-free interval (PFI) than those in the low-expression group (log-rank *p* = 0.00047) (Figure 1B).

For the expression and survival risk, Cox proportional hazards regression analysis was conducted using NTAN1 expression as a continuous variable. The forest plot analysis indicated that higher NTAN1 expression was significantly associated with poorer OS (HR = 2.02, 95% CI: 1.27–3.21, *p* = 0.003) and shorter PFI (HR = 1.59, 95% CI: 1.02–2.48, *p* = 0.04) (Figure 1C,D).

The clinical relevance of NTAN1 protein expression was further assessed using a GBM tissue microarray. The immunohistochemical analysis showed variable NTAN1 staining intensity between the GBM specimens. Representative samples displayed weak NTAN1 staining in the low-expression group and strong staining in the high-expression group (Figure 1E). NTAN1-positive signals were mainly detected in the cytoplasm of tumor cells.

According to the immunohistochemical staining intensity, patients were divided into low- and high-NTAN1 expression groups. Kaplan–Meier analysis demonstrated that patients with high NTAN1 protein expression had significantly shorter overall survival than those with low NTAN1 expression (*p* = 0.0433) (Figure 1F). These data indicate that increased NTAN1 expression is significantly related to unfavorable prognosis in GBM.

### 3.2. NTAN1 Expression Differs Between GBM Cell Lines

**Figure 2:**
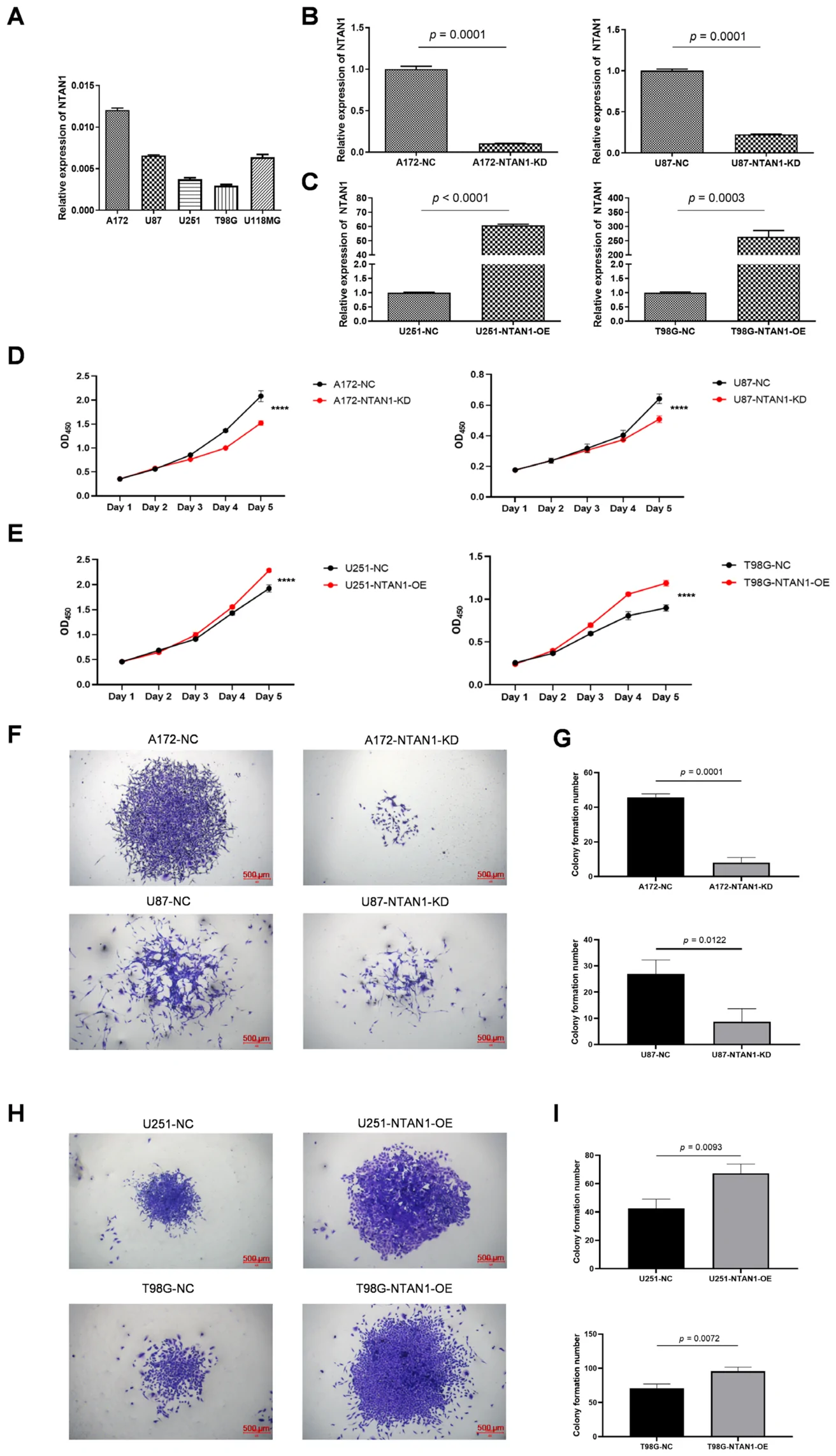
NTAN1 is differentially expressed across GBM cell lines and promotes GBM cell growth. (**A**) Relative NTAN1 mRNA expression levels in a panel of GBM cell lines were determined using qRT-PCR. (**B**) Validation of NTAN1-knockdown efficiency in A172 and U87 cells using qRT-PCR. (**C**) Validation of NTAN1-overexpression efficiency in U251-MG and T98G cells using qRT-PCR. (**D**) CCK-8 assays showing the effects of NTAN1 knockdown on the proliferation of A172 and U87 cells. (**E**) CCK-8 assays showing the effects of NTAN1 overexpression on the proliferation of U251-MG and T98G cells. (**F**) Representative images of colony formation assays in A172 and U87 cells with NTAN1 knockdown. (**G**) Quantification of colony numbers in A172 and U87 cells with NTAN1 knockdown. (**H**) Representative images of colony formation assays in U251-MG and T98G cells with NTAN1 overexpression. (**I**) Quantification of colony numbers in U251-MG and T98G cells with NTAN1 overexpression. Data are presented as the mean ± SD from at least three independent experiments. Statistical significance was analyzed using Student’s *t*-test or two-way ANOVA where appropriate. **** *p* < 0.0001. Scale bar: 500 μm, 4×.

Based on the association between NTAN1 expression and adverse survival outcomes in GBM, endogenous NTAN1 expression was examined in multiple human GBM cell lines. qRT-PCR analysis showed that NTAN1 mRNA expression differed between A172, U87, U251-MG, T98G, and U118-MG cells. A172 cells showed the highest NTAN1 expression, whereas U251-MG and T98G cells displayed relatively low NTAN1 levels. Intermediate NTAN1 expression was observed in U87 and U118-MG cells (Figure 2A). These commonly used human GBM cell lines were selected to represent a panel of GBM models with differing endogenous NTAN1 expression levels, thereby facilitating gain- and loss-of-function analyses.

Accordingly, U251-MG and T98G cells, which expressed low levels of NTAN1, were used for the NTAN1-overexpression experiments. In contrast, A172 and U87 cells, which showed relatively high NTAN1 expression, were selected for the NTAN1-knockdown assays (Figure 2B,C). These expression profiles supported the selection of cell models for subsequent gain- and loss-of-function experiments.

### 3.3. Establishment of Stable NTAN1-Overexpression and Knockdown GBM Cell Models

The efficiency of NTAN1 knockdown and overexpression was confirmed using qRT-PCR. Figure 2B shows that NTAN1knockdown markedly decreased the NTAN1 expression in A172 and U87 cells. Compared with the corresponding control groups, NTAN1 expression was reduced to approximately 10.6% in A172-NTAN1-KD cells and 22.6% in U87-NTAN1-KD cells, with both differences reaching statistical significance (both *p* < 0.001).

Conversely, NTAN1 overexpression substantially increased the NTAN1 expression in U251-MG and T98G cells. Relative to the negative control cells, NTAN1 expression was elevated by approximately 60.7-fold in U251-MG cells and 263.3-fold in T98G cells (both *p* < 0.001) (Figure 2C). These results verified the successful generation of stable NTAN1- knockdown and -overexpression models for functional studies.

### 3.4. NTAN1 Increases GBM Cell Proliferation

After confirming the efficiency of NTAN1 modulation, CCK-8 assays were performed to evaluate the effect of NTAN1 on GBM cell proliferation. NTAN1 knockdown significantly inhibited the proliferation of A172 and U87 cells compared with their respective control cells, as reflected by decreased OD450 values throughout the 5-day observation period (Figure 2D). The suppressive effect became more evident at later time points.

In contrast, NTAN1 overexpression promoted the growth of U251-MG and T98G cells. Cells overexpressing NTAN1 showed higher OD450 values than control cells, especially on days 4 and 5 (Figure 2E). These results demonstrate that NTAN1 enhances the proliferative capacity of GBM cells in vitro.

### 3.5. NTAN1 Strengthens the Colony-Forming Ability of GBM Cells

Colony formation assays were next carried out to examine whether NTAN1 affects the long-term proliferative potential of GBM cells. 2F and 2G show that silencing NTAN1 significantly decreased the number of colonies generated by A172 and U87 cells compared with the corresponding control groups. Colonies formed by NTAN1-knockdown cells were also generally smaller, indicating reduced clonogenic growth.

By contrast, forced NTAN1 expression markedly increased the colony formation in U251-MG and T98G cells. Compared with the control cells, NTAN1-overexpressing cells produced more numerous and larger colonies (Figure 2H,I). These findings indicate that NTAN1 facilitates the clonogenic growth of GBM cells in vitro.

### 3.6. NTAN1 Enhances GBM Cell Migration

**Figure 3:**
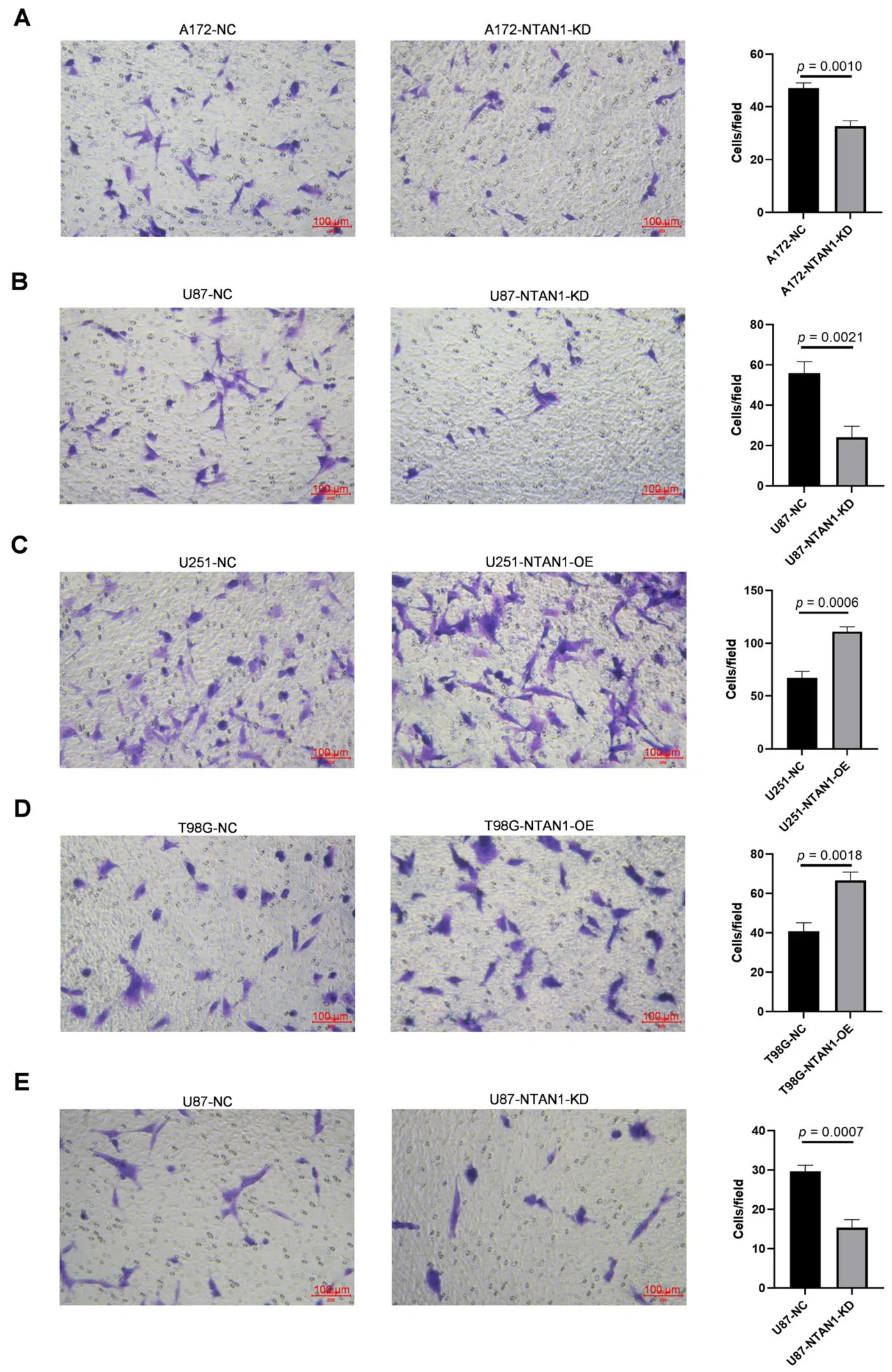
NTAN1 promotes the migratory ability of GBM cells. (**A**) Representative Transwell migration images and quantitative analysis of migrated A172 cells following NTAN1 knockdown. (**B**) Representative Transwell migration images and quantitative analysis of migrated U87 cells following NTAN1 knockdown. (**C**) Representative Transwell migration images and quantitative analysis of migrated U251-MG cells following NTAN1 overexpression. (**D**) Representative Transwell migration images and quantitative analysis of migrated T98G cells following NTAN1 overexpression. (**E**) NTAN1 knockdown suppresses GBM cell invasion in vitro. Representative images (left) and quantification (right) of Matrigel-coated Transwell invasion assays in U87-NC and U87-NTAN1-KD cells. NTAN1 knockdown significantly reduced the number of invading cells (*p* = 0.0007). The number of migrated cells was counted in representative microscopic fields and is presented as the cells per field. Data are shown as the mean ± SD from at least three independent experiments. Statistical significance was determined using Student’s *t*-test. Scale bar: 100 μm, 20×.

The influence of NTAN1 on GBM cell migration was assessed using Transwell migration assays in NTAN1-knockdown and -overexpression models. NTAN1 silencing significantly decreased the number of migrated A172 and U87 cells (Figure 3A,B). In A172 cells, migrated cells were reduced from approximately 47 cells/field in the control group to approximately 33 cells/field after NTAN1 knockdown (*p* = 0.0010, Figure 3A). In U87 cells, NTAN1 knockdown also markedly reduced migration, with migrated cells decreasing from approximately 56 cells/field in the control group to approximately 24 cells/field in the NTAN1-KD group (*p* = 0.0021, Figure 3B).

NTAN1 overexpression significantly increased the migratory ability of U251-MG and T98G cells. The number of migrated U251-NTAN1-OE cells increased from approximately 67 cells/field in the control group to approximately 111 cells/field (*p* = 0.0006, Figure 3C). Similarly, NTAN1 overexpression increased the migrated T98G cells from approximately 41 to 67 cells/field (*p* = 0.0018, Figure 3D). These results show that NTAN1 positively regulates GBM cell migration in vitro.

To further validate the RNA-seq findings indicating that NTAN1 is associated with invasion-related genes and pathways, we performed a Matrigel-coated Transwell invasion assay. NTAN1 knockdown significantly decreased the number of invading U87 cells compared with the control group (*p* = 0.0007; Figure 3E), indicating that NTAN1 contributes to the invasive phenotype of GBM cells in vitro.

### 3.7. NTAN1 Knockdown Decreases the Tumor Volume, Whereas NTAN1 Overexpression Increases the Tumor Volume

**Figure 4:**
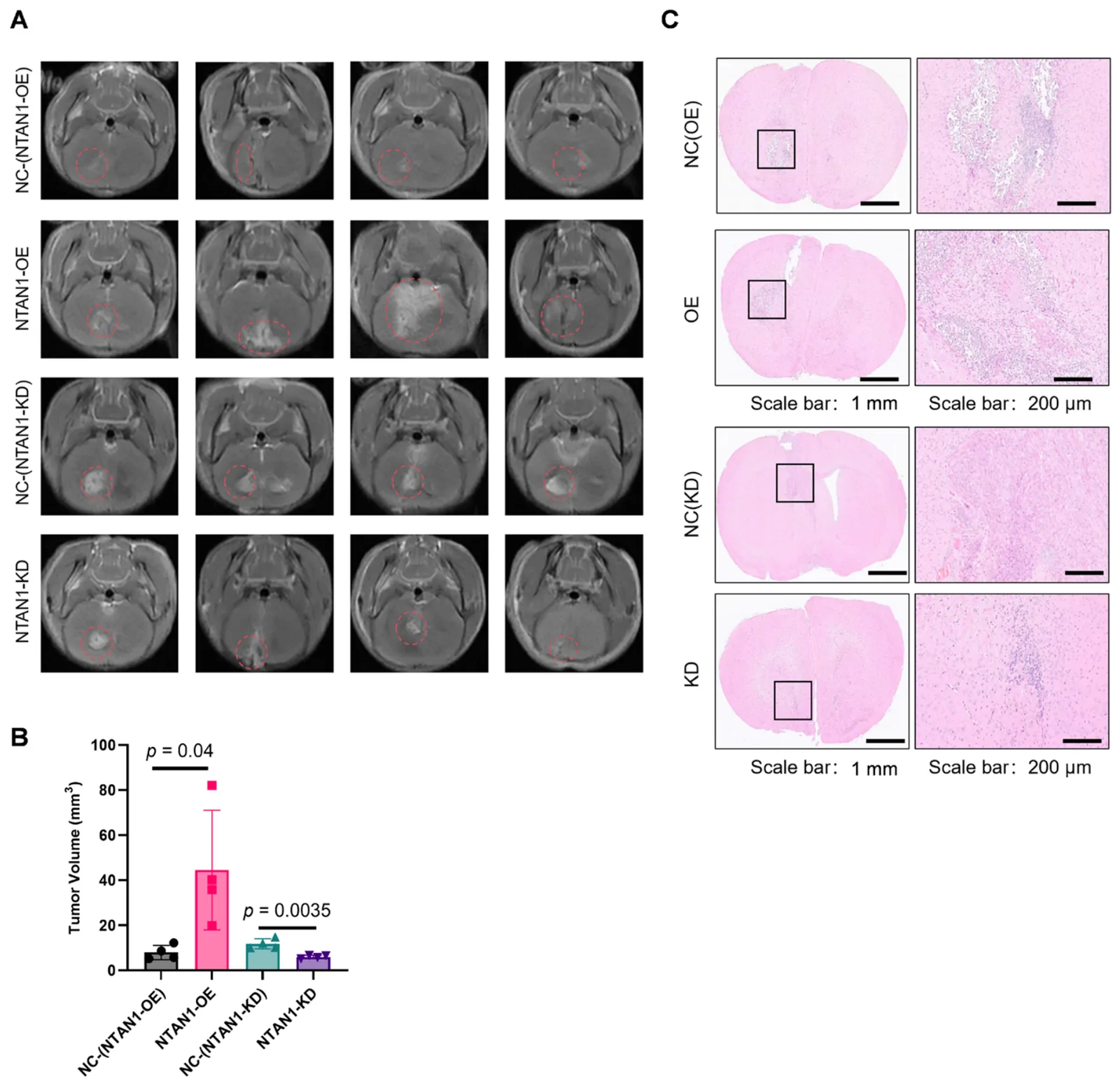
NTAN1 promotes GBM tumor growth in vivo. (**A**) Representative magnetic resonance imaging (MRI) images showing tumors derived from U87 control, NTAN1-knockdown, and NTAN1-overexpressing cells in nude mice. (**B**) Quantitative analysis of tumor volume at the experimental endpoint. Tumors derived from NTAN1-knockdown (KD) cells showed significantly reduced tumor volumes, whereas tumors from NTAN1-overexpressing (OE) cells exhibited significantly larger tumor volumes compared with the respective control groups. (**C**) Representative hematoxylin and eosin (H&E) staining images of tumor tissues from each group, verifying the pathological characteristics of xenograft tumors. Data are presented as the mean ± SD. Statistical significance was determined using one-way ANOVA, followed by Tukey’s post-test.

To evaluate the effect of NTAN1 on GBM growth in vivo, subcutaneous xenograft models were generated by injecting U87 control cells, NTAN1-knockdown cells, or NTAN1-overexpressing cells into nude mice. Figure 4A,B show that tumors formed by NTAN1-knockdown (KD) cells exhibited slower growth and smaller volumes than tumors derived from control cells (NC) and NTAN1-overexpressing (OE) cells. The tumor volume was significantly increased in the NTAN1-OE group compared with the control group (*p* = 0.04), whereas the tumor volume was significantly decreased in the NTAN1-KD group compared with the NC group (*p* = 0.0035).

Hematoxylin and eosin (H&E) staining further showed differences in tumor progression between the groups. Tumors from the NTAN1-knockdown group displayed reduced proliferation, whereas tumors from the NTAN1-overexpressing group showed enhanced growth and increased cellular density (Figure 4C).

These in vivo data were consistent with the in vitro findings that NTAN1 promotes GBM cell proliferation and colony formation.

### 3.8. NTAN1 Knockdown Reduces Multiple Classical Factors Involved in Cancer Invasive Growth

**Figure 5:**
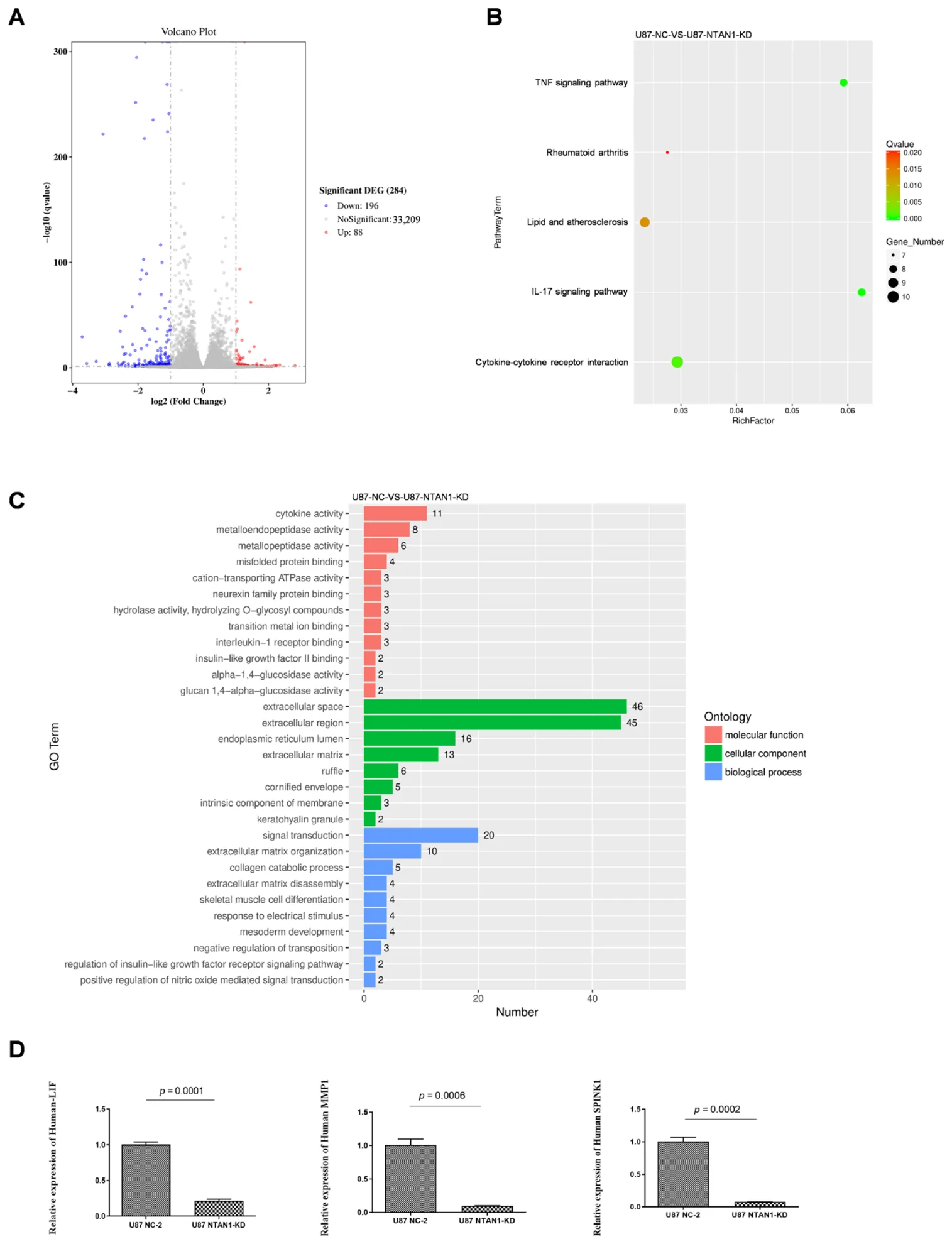
Transcriptomic analysis reveals that NTAN1 regulates invasion-related genes in GBM cells. (**A**) Volcano plot showing differentially expressed genes in U87 cells following NTAN1 knockdown compared with control cells. A total of 284 significantly altered genes were identified, including 196 downregulated genes and 88 upregulated genes. (**B**) KEGG pathway enrichment analysis of differentially expressed genes after NTAN1 silencing, showing enrichment in the cytokine–cytokine receptor interaction, IL-17 signaling pathway, TNF signaling pathway, and other related pathways. (**C**) GO enrichment analysis of differentially expressed genes, highlighting biological processes, cellular components, and molecular functions associated with extracellular matrix remodeling, collagen catabolic process, metallopeptidase activity, and cytokine activity. (**D**) RT-qPCR validation of representative invasion-associated genes, including *SPINK1*, *MMP1*, and *LIF*, in U87 cells with or without NTAN1 knockdown. Data are presented as the mean ± SD from at least three independent experiments. Statistical significance was determined using Student’s *t*-test. Exact *p* values are indicated in the panels.

To investigate the molecular changes associated with NTAN1 in GBM, transcriptome sequencing was performed in U87 cells with or without NTAN1 knockdown. The differential expression analysis identified 284 significantly altered genes after NTAN1 silencing, including 196 downregulated genes and 88 upregulated genes (Figure 5A).

Enrichment analyses were then conducted to characterize the biological functions of these differentially expressed genes. KEGG pathway analysis revealed that the altered genes were mainly enriched in cytokine–cytokine receptor interaction, IL-17 signaling, and TNF signaling pathways (Figure 5B). GO enrichment analysis further showed a significant enrichment of terms related to extracellular matrix disassembly, collagen catabolic process, extracellular matrix organization, metallopeptidase activity, metalloendopeptidase activity, and cytokine activity (Figure 5C).

To confirm the RNA-seq results, three representative invasion-associated genes, *SPINK1*, *MMP1*, and *LIF*, were selected for RT-qPCR validation. Consistent with the transcriptome sequencing data, NTAN1 knockdown significantly decreased the expression of *SPINK1* (*p* = 0.0002), *MMP1* (*p* = 0.0006), and *LIF* (*p* = 0.0001) (Figure 5D). These results indicate that NTAN1 knockdown reduces the expression of genes associated with extracellular matrix remodeling, cytokine signaling, and cancer invasive growth.

Overall, these data showed that NTAN1 expression was significantly up-regulated in GBM and was associated with poor prognosis. In addition, NTAN1 promoted GBM progression through a series of classical factors involved in the invasive growth of cancer cells.

## 4. Discussion

The diffuse and highly infiltrative growth pattern of glioblastoma remains a major challenge in clinical management, as it limits complete surgical resection and contributes to a nearly inevitable recurrence. Although this feature is central to GBM aggressiveness, the molecular basis underlying tumor migration and infiltrative growth has not been fully defined. In the present study, we identified NTAN1 as a clinically relevant factor in GBM. Increased NTAN1 expression was associated with unfavorable survival in patients with GBM, and this finding was supported by bioinformatics analysis and the immunohistochemical evaluation of tumor tissues. These data suggest that NTAN1 may serve as a potential prognostic biomarker in GBM.

Our functional results further indicate that NTAN1 is not only associated with aggressive disease but also contributes to GBM progression. NTAN1 knockdown inhibited GBM cell proliferation, clonogenic growth, migration and invasion, whereas NTAN1 overexpression promoted tumor growth and migratory potential in vitro and in vivo. The consistency between the loss- and gain-of-function experiments supports a tumor-promoting role for NTAN1. In addition, the orthotopic xenograft model suggests that the biological effects of NTAN1 are maintained in a physiologically relevant intracranial setting. However, although the in vivo model demonstrated a clear effect on tumor growth, the precise contribution of NTAN1 to infiltrative behavior within the brain microenvironment remains to be further defined.

Mechanistically, NTAN1 encodes N-terminal asparagine amidase 1, a component of the N-degron pathway involved in selective protein degradation. Although its role in GBM has not been previously characterized, dysregulated protein turnover has been increasingly recognized as an important feature of malignant progression. Our findings therefore raise the possibility that NTAN1 may promote GBM growth and invasiveness by affecting degradation-dependent regulatory networks. While the direct substrates of NTAN1 in GBM remain unknown, the present data suggest that its effects are likely mediated through broader cellular programs rather than a single downstream target.

Previous studies have linked NTAN1 to molecular abnormalities in pediatric cancers [20,21]. In addition, NTAN1 has been reported as part of a cancer-associated fibroblast-related gene signature in gastric cancer [22], suggesting a broader role in tumor progression. Consistent with these observations, our RNA-seq data provide an important framework for understanding the phenotypic effects of NTAN1 in GBM. Among the downregulated genes following NTAN1 knockdown, *SPINK1*, *MMP1*, and *LIF* are of particular interest because they have been associated with tumor aggressiveness, extracellular matrix remodeling, inflammatory signaling, and invasive growth in multiple malignancies, including GBM-related contexts [23,24,25]. *MMP1* may contribute directly to matrix degradation and tissue invasion, whereas *LIF* has been implicated in tumor cell plasticity and microenvironmental signaling. *SPINK1* has also been linked to aggressive biological behavior in several cancers. Although our study does not establish a direct mechanistic hierarchy between NTAN1 and these genes, these transcriptomic changes are consistent with the observed inhibitory effects of NTAN1 silencing on GBM cell migration and Matrigel invasion.

Pathway enrichment analysis further identified cytokine–cytokine receptor interaction, IL-17 signaling, TNF signaling, extracellular matrix organization, extracellular matrix disassembly, and metallopeptidase-related activity as pathways altered after NTAN1 knockdown. Although these findings are correlative, they are biologically plausible in the context of GBM. IL-17 and TNF signaling have been linked to inflammatory responses, NF-κB activation, cytokine production, extracellular matrix remodeling, and enhanced tumor cell motility. In glioma, inflammation-associated signaling is increasingly recognized as a contributor to invasive behavior and tumor–microenvironment crosstalk. Therefore, the enrichment of these pathways suggests that NTAN1 may influence GBM progression, at least in part, through inflammation-related and extracellular remodeling-associated molecular networks. However, further mechanistic studies will be needed to determine whether these pathways are directly involved in NTAN1-mediated phenotypes.

Several limitations should be noted. Although the prognostic relevance of NTAN1 was supported by public datasets and tissue-based validation, confirmation in larger independent cohorts is still needed. In addition, while transcriptomic analysis identified candidate downstream mediators, the direct molecular mechanism by which NTAN1 promotes GBM progression was not established in this study. Moreover, although the orthotopic model demonstrated an effect on tumor growth, further work is required to define how NTAN1 influences infiltrative behavior in vivo. Validation in patient-derived models would also strengthen the biological and translational relevance of these findings.

In summary, our study identifies NTAN1 as a novel contributor to GBM progression. Elevated NTAN1 expression is associated with poor prognosis, and functional analyses indicate that NTAN1 promotes GBM cell proliferation, clonogenic growth, migration, and in vivo tumor growth. These findings suggest that NTAN1 may serve as a promising prognostic biomarker and potential therapeutic target in GBM.

## Data Availability

The data presented in this study are available on request from the corresponding author due to privacy and ethical restrictions.
